# Subjective Social Status Is Associated with Dysregulated Eating Behaviors and Greater Body Mass Index in an Urban Predominantly Black and Low-Income Sample

**DOI:** 10.3390/nu13113893

**Published:** 2021-10-29

**Authors:** Monika M. Stojek, Paulina Wardawy, Charles F. Gillespie, Jennifer S. Stevens, Abigail Powers, Vasiliki Michopoulos

**Affiliations:** 1 Department of Social Sciences, University of Silesia, 40-007 Katowice, Poland; paulina.wardawy@us.edu.pl; 2 Department of Psychiatry and Behavioral Sciences, Emory University School of Medicine, Atlanta, GA 30329, USA; cgilles@emory.edu (C.F.G.); Jennifer.stevens@emory.edu (J.S.S.); adpower@emory.edu (A.P.); vmichop@emory.edu (V.M.); 3 Yerkes National Primate Research Center, Emory University, Atlanta, GA 30322, USA

**Keywords:** obesity, food addiction, emotional eating, subjective social status, health inequities

## Abstract

Background: Higher subjective social status (SSS) or a person’s perception of their social standing is related to better health outcomes, but few studies examined SSS in relation to obesity. Emotional eating and food addiction have been linked to obesity. Some studies indicated that manipulating SSS may lead to altered food intake, but the relationship between SSS and dysregulated eating, such as emotional eating and food addiction (FA), has not been examined. The goal of this study was to examine the associations between SSS in the community and the larger society, dysregulated eating (emotional eating and FA), and body mass index (BMI) in a majority racial minority sample. Methods: The participants (*N* = 89; 93% Black, 86% women, and 56% with obesity; 72% income lower than USD 2000), recruited from a publicly funded hospital in Atlanta, GA, completed the MacArthur Scale, Dutch Eating Behaviors Questionnaire, Yale Food Addiction Scale, Beck Depression Inventory, PTSD Symptom Checklist, and demographics questionnaire. Results: Twenty-two percent of the sample met the criteria for FA; those with FA had significantly higher BMI than those without (*p* = 0.018). In the hierarchical linear regression, the SSS community (but not in society) predicted higher severity of emotional eating (*β* = 0.26, *p* = 0.029) and FA (*β* = 0.30, *p* = 0.029), and higher BMI (*β* = 0.28, *p* = 0.046), independent from depression and PTSD symptoms. Conclusions: The findings indicate that, among Black individuals with predominantly low income in the U.S., perceived role in their community is associated with eating patterns and body mass. Given the small sample size, the results should be interpreted with caution.

## 1. Introduction

Subjective social status (SSS) refers to a person’s perception of their social standing in the community and society [1]. Individuals who perceive themselves as having higher social standing in their society and community tend to report better physical and mental health [2,3,4]. SSS is distinct from objective social status, measured by indicators such as income or education [5], and predicts health separately from the objective social status indicators [5,6,7]. SSS captures the psychological variables associated with social standing, such as respect and influence in face-to-face groups, perceived prospects for social mobility, and internalization of the subordinate status [8], rather than the objective indicators of socioeconomic status that reflect a person’s position in the social hierarchy at one point in time [9]. That may explain why SSS is more strongly associated with health indicators than objective social status. Some have suggested that the perception of inequality accompanied by “psychological pain” rather than objective socioeconomic resources is what adversely impacts health [10]. Others suggested that the internalization of subordinate status may be what negatively impacts health [11]. Therefore, SSS may help explain how social inequality affects health. This seems particularly relevant, as a recent meta-analysis concluded that SSS and objective SES are more closely associated in White samples while the perception of social standing may be less predicated on the availability of objective resources in Black samples [7]. Thus, it is particularly important to examine SSS and its effect on health in minority samples.

The obesity epidemic [12] is one of the greatest challenges for global public health [13], as obesity is associated with a number of adverse health outcomes, including coronary heart disease; type 2 diabetes mellitus [14,15]; and increased mortality [16], including from COVID-19 [17,18]. In adolescent samples, lower SSS was associated with overweight and obesity cross-sectionally [19,20,21] and longitudinally [22]. There is a dearth of studies examining SSS in relation to obesity in adults. One large study in British older adults found no relationship between SSS and central obesity (i.e., waist circumference) after adjusting for covariates [11]. One study in healthy White women examined SSS in the society (but not in the community) in relation to body mass index (BMI) and found no significant relationship. To our knowledge, no studies have examined SSS in relation to weight and factors that contribute to weight, including dysregulated eating behavior, in Black adults. It is an important gap in research as close to 60% of Black women and over 40% of Black men in the U.S. have obesity; additionally, nearly 20% of Black women and nearly 10% of Black men have severe obesity (i.e., BMI ≥ 40) [23]. However, it is unclear whether past findings on SSS and obesity are generalizable to Black Americans. 

Dysregulated eating patterns, such as emotional eating and food addiction, have been identified as likely contributing to the obesity epidemic in the past several decades with the increased availability of highly processed high-fat, high-sugar foods [24,25,26]. Emotional eating refers to eating in response to negative emotions or stress [27,28] and has been linked to increased BMI in adult, predominantly female European samples [29], [30]. Emotional eating also predicted weight and adiposity increases over one year in adolescents who reported episodes of loss of control eating at baseline [31]. One study in a primarily Black sample of women found a positive association between emotional eating and BMI [32]. Food addiction (FA) describes a group of dysregulated eating behaviors that are phenomenologically similar to behaviors conventionally associated with substance dependence, such as eating larger amounts or for longer periods of time than planned, binge eating, tolerance to certain foods, and persistent and unsuccessful attempts to cut down on certain foods [33]. FA is more prevalent among patients with obesity than without [34,35] and has been associated with BMI in many [36,37,38,39], but not all (for a review, see [25]), studies. 

There is some evidence to indicate that internalization of the subordinate social status may be a psychosocial stressor that contributes to dysregulated eating patterns. A small pilot study that manipulated participants’ (young adult Latino women and men) perceived social standing in a game of rigged Monopoly^TM^ found that participants in the low social status condition consumed a significantly higher proportion of their daily calorie needs relative to the high social status condition in an ad libitum buffet lunch [40]. A series of four studies among adults in Singapore demonstrated that those who were experimentally induced to feel socially subordinate demonstrated greater preference for and intake of high-calorie foods [41]. When placed in the low social status condition, participants also reported decreased perceptions of powerfulness and pride, compared with when they were in the high social status condition [40]. Lower SSS was also found to be associated with greater post-lunch energy intake in an 14-day observational study, indicating poorer energy compensation following a large meal among individuals who perceive themselves as subordinate in their society [42]. Such deficits in energy compensation may result in excessive weight gain over time, although that study did not test such effects over time [42]. In a large survey study of college students, lower SSS was related to poorer emotion regulation skills, which in turn were related to greater expectancies that food would help manage negative affect and alleviate boredom [43]. One experimental study exploring the mechanisms of the relationship between perceived social status and caloric intake found that inducing social subordination elicited social anxiety, which in turn led to increased caloric intake among people who reported a strong need to belong [44]. While there is experimental evidence that SSS may lead to altered food intake, its relationship to dysregulated eating patterns such as emotional eating and food addiction has not been examined. 

It is important to account for the effects of trauma and depression when investigating dysregulated eating. The experience of trauma and the possible resulting symptoms (posttraumatic stress disorder or PTSD) have been linked to dysregulated eating patterns [45,46]. In fact, it has been proposed that changes in the sympathetic nervous system and the neuroendocrine system that occur as a result of PTSD symptomatology represent a metabolic disorder and, as such, substantively contribute to weight gain and obesity [47]. Dysregulated eating, including emotional eating and FA, has also been associated with elevated depression levels [32,45]. 

While the associations between lower SSS, obesity, and eating behavior are documented, relatively little is known about these associations in samples who experience objective social disadvantage. Additionally, the relationship of SSS to emotional eating and food addiction, two dysregulated eating patterns that may lead to weight gain over time, has not been studied. The aim of this study was to examine the relationship between SSS, dysregulated eating behaviors, and body mass index (BMI) in a majority racial minority sample. We hypothesized that lower SSS, both in the community and in the society, is associated with greater severity of emotional eating and food addiction. We also hypothesized that SSS is significantly related to BMI, such that individuals with higher perceived social status demonstrate lower BMI. 

## 2. Materials and Methods

### 2.1. Participants and Procedures

Participant recruitment took place in a publicly funded hospital in Atlanta, Georgia, from the waiting rooms in diabetes, gynecology, and primary care medical clinics. To participate in the study, participants had to be between the ages of 18 and 65, be able to speak English, and have an actual phone number for possible contact with them. Data were obtained only from participants who gave their informed consent. 

Procedures for the current study included participation in a screening interview and completing the questionnaires selected for the research study. The screening interview either took place in the waiting room of the medical clinic or in the research laboratory by trained research assistants who read aloud assessments on trauma and psychological symptoms. If participants began the interview in the waiting room but were called back for their appointment, the additional questionnaires were finished in a research laboratory visit. The participants were compensated USD 15 for visits in the waiting room and USD 20 for visits in the research laboratory (to cover additional costs for travel). Research procedures were approved by the Emory Institutional Review Board and the Grady Hospital Research Oversight Committee.

### 2.2. Measures

#### 2.2.1. Subjective Social Status

Subjective social status was assessed using The MacArthur Scale of Subjective Social Status [48]. The tool measures perceived placement within the social hierarchy using a visual scale. Participants place themselves on a 10-rung ladder [1]. The top of the scale represents people with the most money, respected jobs, and education. The bottom depicts people with the least money, least respected jobs, and least education. The scale is reverse-scored: therefore, individuals who rate their SSS as the highest on the ladder receive a score of “1” and individuals who rate their SSS as the lowest on the ladder receive a score of “10” [1]. The scale is available in two versions: the community ladder and the society ladder. The community ladder assesses the hierarchical placement of a self-defined social group. It refers to a local, nearest social environment. Society ladder assesses placement in the social hierarchy compared with other people in society, and it is associated with traditional SES indicators. We included both the community and the society ladders in our study.

#### 2.2.2. Eating Dysregulation

The severity of emotional eating was assessed with the Emotional Eating subscale of the Dutch Eating Behaviors Questionnaire (DEBQ) [30]. The Emotional Eating subscale comprises 13 items (e.g., “Do you have the desire to eat when you are irritated?”) on a 5-point scale from 0 (*No desire*) to 4 (*A strong desire*). The items measure desire to eat under various negative emotional states, such as disappointment, irritation, and loneliness. THe internal consistency of this measure in our sample was α = 0.95.

Food addiction was measured with the Yale Food Addiction Scale (YFAS) [33]. The YFAS is composed of 25 items and assesses addictive eating behavior, for instance, persistent desire or repeated unsuccessful attempts to quit (“I want to cut down or stop eating certain kinds of food”) and food tolerance (“I have found that eating the same amount of food does not reduce my negative emotions or increase pleasurable feelings the way it used to”). The measure has two scoring methods. First, there is a continuous scoring method that summarizes how many of the 11 SUD criteria an individual endorsed with respect to consuming highly palatable foods. This method was used to generate FA severity in our sample. Second, the measure is scored to assess a “diagnostic” threshold, which is met if an individual endorses three or more symptoms plus impairment or distress. That method was used to generate the FA status in our sample. The internal consistency of this measure in our sample was α = 0.90.

#### 2.2.3. Body Mass Index (BMI)

BMI was calculated using participants’ self-reported height and weight. While self-reported BMI tends to be lower compared with the measured BMI, the correlations of self-reported and measured BMI values are very high (Pearson’s *r* correlations above 0.90), and they are equally related to biomarkers of obesity [49]. BMI was calculated using a standard equation ((lbs × 703)/inches squared). Based on the BMI, weight status was coded as 1 = normal weight (18.5 < BMI < 25), 2 = overweight (25 < BMI < 30), 3 = obese (BMI > 30), and 4 = underweight (BMI < 18.5); the cut-offs used were those recommended by the World Health Organization [50].

#### 2.2.4. Objective Socioeconomic Status

Participants reported the following objective socioeconomic status (SES) indicators in the demographics form: monthly income (0 = USD 0–USD 249, 1 = USD 250–USD 499, 2 = USD 500–USD 999, 3 = USD 1000–USD 1999, and 4 = USD 2000 or more), level of education (0 = less than 12th grade, 1 = 12th grade/high school graduate, 3 = some college or technical school, 4 = technical school graduate, 5 = college graduate, and 6 = graduate school), and employment status (0 = unemployed and 1 = employed). 

#### 2.2.5. Demographics

Participants completed a demographic questionnaire in which they identified their binary sex (0 = male and 1 = female), age (in years), race (0 = Black, 1 = White, and 2 = all other races), ethnicity (0 = Hispanic/Latinx and 1 = not Hispanic/Latinx), disability status (0 = no disability and 1 = positive disability status), and relationship status (0 = single, never married, 1 = married, 2 = divorced, 3 = separated, 4 = widowed, and 5 = domestic partner). 

#### 2.2.6. Covariates

*PTSD Symptom Severity*. As current and past PTSD symptoms are associated with dysregulated eating [45,51] and BMI [47,52], we included PTSD symptom severity in the initial correlational analysis as a possible covariate, where associations with outcome variables were found. The severity of PTSD symptoms was assessed using the PTSD Symptom Checklist for DSM-5 (PCL-5) [53]. The PCL-5 comprises 20 items to which the participants respond by choosing an answer on a 5-point scale (0—not at all; 4—extremely). This self-report scale measures the severity of PTSD symptoms in the past month. Higher scores indicate greater severity of symptoms. The proposed cut-off score for a possible PTSD diagnosis is between 31 and 33 [54]. The internal consistency of this measure in our sample was α = 0.94.

*Depression Symptoms Severity*. Given that, in past studies, elevated depression was associated with emotional eating, as measured by the DEBQ [32], we included depression measurement in the initial correlation analyses as a possible covariate. The severity of depression symptoms was measured using The Beck Depression Inventory (BDI-II) [55]. The BDI-II is a self-report scale with 21 items. The respondents assess the severity of depression symptoms in the last two weeks on a 4-point scale, ranging from 0 to 3. The total score is the sum of all responses, with a possible total score of 63. Higher scores indicate greater severity of depression symptoms, with scores >18 indicative of likely clinical levels of depression. The internal consistency of this measure in our sample was α = 0.93.

### 2.3. Statistical Analyses

We examined the data for normality and missingness. We conducted bivariate correlation analyses to examine the interrelationships between SSS (community and society) and outcome variables (FA severity, emotional eating severity, and BMI). In order to identify potential covariates in each regression model, we conducted bivariate Pearson correlation analyses between the outcome variables (FA severity, emotional eating, and BMI) and PTSD and depression symptoms (measured by the PCL-5 and the BDI-II, respectively) as well as objective measures of SES (level of education, current employment, and monthly income). The variables that were significantly correlated with the outcome variables at *p* < 0.05 were included in that regression model. The covariates included depression severity (emotional eating and FA models), PTSD severity (FA model), BMI (emotional eating and FA models), FA status (BMI model), and sex (BMI model); see the Results section for the correlation coefficients.

Hierarchical linear regressions were conducted to examine the relative contributions of each aspect of the SSS to each of the outcomes of interest (emotional eating, FA, and BMI), while statistically adjusting for covariates. In the first model, we entered the scores on the DEBQ emotional eating scale as the outcome variable, covariates in the first step of the model, and SSS community and society in the second step of the model. In the second model, we entered FA severity as the outcome variable, covariates in the first step of the model, and SSS community and society in the second step of the model. In the third model, we entered BMI as the outcome variable, covariates in the first step of the model, and SSS community and society in the second step. 

All analyses were conducted using SPSS version 27. An alpha level of *p* ≤ 0.05 was considered statistically significant; as the three hypotheses were made a priori, no corrections were applied to the analyses.

## 3. Results

### 3.1. Sample Characteristics

A total of *N* = 89 participants completed the SSS and the DEBQ, and *n* = 68 were administered the YFAS (the YFAS was administered at the end of the larger battery of surveys). We examined the patterns of missingness as recommended [56,57] and determined that the participants who completed the YFAS did not significantly differ from those who did not (*p*-values ranging from 0.230 to 0.971). We concluded that data were missing completely at random (MCAR) and that pairwise deletion was employed in the analyses with the YFAS. Therefore, the sample sizes for the regression analyses vary and are reported separately for each analysis.

The demographic characteristics of the sample are reported in Table 1. Individuals in the sample were predominantly women and predominantly Black, and the majority of them (56%) had obesity, with the average BMI also in the obese range (M = 32.66, SD = 9.11). Twenty-two percent of the sample had a positive FA status. A one-way analysis of variance test indicated that individuals with the FA status had a significantly higher BMI (M = 37.94, SD = 8.07) compared with individuals without FA (M = 31.50, SD = 8.98; F = 5.92, *p* = 0.018). The mean PCL score (M = 39.84; SD = 20.78) was above the suggested cutoff of 33 [54], indicating a highly traumatized sample with a possible PTSD diagnosis in 63% of participants. The mean BDI score (M = 25.40, SD = 14.08) suggested a sample with clinical levels of depression in 70% of the sample. 

### 3.2. Correlations between Perceived Social Status vs. Objective Indicators of Socioeconomic Status and Variables of Interest

The correlation between SSS society and community was *r* = 0.46 (*p* < 0.001). The results of bivariate Pearson correlations between SSS (both in the society and the community) and other variables of interest are reported in Table 2. Overall, lower SSS society and community was associated with higher FA severity (*p* < 0.01) and with greater severity of emotional eating (*p* < 0.05 for society; *p* < 0.05 for community). Lower SSS in the community, but not in society, was associated with higher BMI (*p* < 0.01). In contrast, none of the objective SES indicators (i.e., level of education, employment status, or income level) were significantly associated with dysregulated eating or BMI (see Table 3). Therefore, the objective indicators of SES were not included in the regression models as covariates.

### 3.3. Associations between Perceived Social Status and Eating Dysregulation

We examined the relative contributions of SSS in the society and community on eating dysregulation using two hierarchical linear regression models. In the model with DEBQ emotional eating scale as the outcome variable, we entered the following covariates in the first step: depression severity as it was significantly associated with DEBQ severity in bivariate correlations (r = 0.262, *p* = 0.013) and BMI to statistically adjust for the effects of weight. In the second step of the model, we entered the scores on the society and community social ladder. We found that SSS community, but not society, contributed significant variance to the prediction of emotional eating (*p* = 0.029; see Table 4). In the second model, we entered FA symptoms severity as the outcome variable. In the first step of the model, we entered BMI to statistically adjust for the effect of weight. Additionally, FA severity was correlated with PTSD severity (r = 0.247, *p* = 0.043) and depression severity (r = 0.320, *p* = 0.008), therefore, we entered these variable in step 1 as covariates to statistically adjust for their effects. In step 2, we entered the scores on the society and community social ladders. We found that perceived social status in the community, but not in the society, contributed significant variance to the prediction of FA severity (*p* = 0.029; see Table 5).

### 3.4. Associations between Perceived Social Status and BMI

We examined the relative contributions of SSS on BMI using a hierarchical linear regression. In step 1, we statistically adjusted for the effects of sex; additionally, as FA status was significantly correlated with BMI (r = 0.289, *p* = 0.018), we adjusted for FA status (positive vs. negative) in step 1 as well. In step 2, we entered the scores on the society and community ladders. We found that social status in the community, but not in the society, contributed significant variance to the prediction of BMI (*p* = 0.046; see Table 6). 

## 4. Discussion

The present study aimed to examine the contributions of perceived social status in the society and community on dysregulated eating behavior and BMI in a majority racial minority sample. We found that, in a sample of predominantly Black women, subjective social status in the community, but not in society, was associated with emotional eating, FA symptoms, and BMI. Specifically, people who perceived themselves as having higher standing in their community reported lower severity of dysregulated eating as well as lower BMI. Contrary to our hypotheses, we found no relationships between subjective social status in the society and dysregulated eating or BMI. Of note, we found relatively high rates of FA and obesity in the current sample, with individuals with positive FA status demonstrating significantly higher BMI compared with those without FA. The study was conducted in a sample of predominantly Black and predominantly low-income city dwellers (primarily women) seeking medical care at a local publicly funded hospital. Therefore, its findings are generalizable to similar populations and shed light on the association between low subjective social status and dysregulated eating behaviors. 

While the finding that subjective social status (SSS) in the society is not related to dysregulated eating or BMI is contrary to our hypotheses, it is not completely unexpected. The evidence for the link between SSS in society and obesity appears to be mixed, with some [19,22] but not all [20,21] studies in adolescent samples demonstrating a link between SSS in society and obesity. In fact, in one study that demonstrated such link, SSS in the community was more strongly associated with health than SSS in the society when both were included in the model [19]. Thus, even among adolescents, social standing in the community (measured in adolescents as their social standing at school) appears more important in predicting health than the SSS in the society [20,21]. Among adults, SSS in the society has not been associated with body mass, neither among older British adults [11] nor in a sample of healthy White women [1]. Our results in a sample of majority Black women support past findings in other samples indicating that social status in the greater society does not appear to be as impactful on body mass as social status in the more immediate local community. This finding is consistent with Bonfenbrenner’s socioecological model, suggesting that the status in the community is more salient than the status in the society because of the immediate context of our interactions with those in our community [58]. The current study is the first study to demonstrate that higher social status in the community but not in society is associated with lower emotional eating and fewer FA symptoms, pointing to a potential mechanism of action between perceptions of subordinate social status in one’s community and increased body weight. One possible mechanism that considers the environment is that individuals lower in the social hierarchy have access to fewer social resources and experience more social unpredictability, further contributing to chronic stress [59]. Past studies indicated that unpredictability schemas, including those related to neighborhood quality, contribute to dysregulated eating [60]. Another potential explanation accounts for neurobiological pathways involved in consuming high-calorie foods. Animal studies have demonstrated that psychosocial stress related to subordinate status in a social hierarchy is associated with increased caloric intake in environments with high availability of such foods [61,62]. The relationship between stress and dysregulated eating appears to be mediated by alterations in neurobiology of the corticostriatal-reward pathways [63], including the “reward deficiency syndrome,” characterized by reduced dopaminergic activity [64]. These stress-induced neurobiological changes have been linked to “comfort food” ingestion in stressful situations, contributing to obesity, in animal studies [65,66]. Thus, it appears that social regulation of eating behavior may be evolutionarily conserved. In humans, several experimental studies demonstrated that inducing subordinate status affected participants’ eating preferences and caloric intake, such that they tended to eat more or choose higher-calorie foods than participants in high social status conditions [40,41,44]. A study that measured energy intake over a two-week period indicated that SSS in the society (SSS in the community was not measured) was associated with greater energy intake after lunch [42]. Another study among college students indicated that lower SSS in the community was indirectly associated with expectations that food would help with emotion regulation through poorer emotion regulation skills [43]. Our study adds to this body of work, pointing to the importance of one’s standing in the community, particularly among Black women, to their eating patterns and body mass. It appears that a favorable social status in the immediate community may contribute to more effective emotion regulation skills (as demonstrated by lower emotional eating among participants with higher SSS) and fewer problems related to eating (as demonstrated by lower food addiction scores among participants with higher SSS). This in turn may lead to lower body mass, but that is an empirical question—although we found that higher SSS in the community is associated with lower BMI, we did not test the mechanistic relationship. These findings also suggest that it is important to understand an individual’s perception of their social standing, especially in the their proximal social context, when intervening in dysregulated eating or weight loss therapy goals. If higher social status is indeed associated with more effective emotion regulation skills, then applying certain cognitive-behavioral strategies (e.g., checking the facts of the situation and problem solving around more effective emotion regulation responses) specific to situations when an individual’s feeling of social inferiority is activated may be an important part of the treatment plan addressing dysregulated and weight loss.

The strengths of the current study included participation of an underrepresented population of Black women with high rates of history of trauma. Including more women, particularly of racial minorities, is a priority for research. Showing that individuals who experience objective social disadvantage demonstrate similar patterns of relationships between SSS and physical health as other samples points to the generalizability of these findings and to potential intervention points. We also investigated SSS in the community, an understudied construct in past research [7], and demonstrated that it is more instrumental to weight-related health and behaviors than SSS in the society. However, the study is not without limitations. The limitations of this study include a small sample size; therefore, the results should be interpreted with caution. The small sample size also precluded us from testing a mediational relationship between SSS in the community and body mass via dysregulated eating. We also were unable to examine sex differences given a small proportion of men in the sample. The study should be replicated in a larger sample of both women and men. Future studies would benefit from examining the relationship between SSS and obesity longitudinally to test the potential mediating effect of dysregulated eating patterns. 

## 5. Conclusions

The current study provides preliminary evidence that one’s perceived role in their community has a role in their health outcomes among Black women. It also points to potential intervention targets around a person’s perception of their standing in their community when addressing dysregulated eating and weight loss. Importantly, this is the first study examining SSS in a sample of individuals who experience objective economic disadvantage. While the findings should be replicated in a larger sample, they add to our understanding on economic health inequities.

## Figures and Tables

**Table 1:** Demographic and clinical characteristics of the participants who completed the MacArthur Scale of Subjective Social Status.

		%/Mean (SD)
Sex	Women	86.3%
	Men	13.7%
Age		45.7 (11.5)
Race	Black	92.7%
	White	1%
	Other Races	6.3%
Ethnicity	Hispanic/Latinx	2.1%
	Non-Hispanic/Latinx	97.9%
Education	Less than high school	15.6%
	High school	26.0%
	GED	3.1%
	Some college or technical school	31.3%
	College graduate	13.5%
	Technical school graduate	5.2%
	Graduate school	5.2%
Employment status	Employed	33.7%
	Unemployed	66.3%
Disability status	Receiving disability	30.9%
	Not receiving disability	69.1%
Monthly income	USD 0–249	7.6%
	USD 250–499	9.8%
	USD 500–999	16.3%
	USD 1000–1999	38.0%
	USD 2000 or more	28.3%
Relationship status	Single	49.5%
	Married	14.7%
	Divorced	16.8%
	Separated	5.3%
	Widowed	8.4%
	Living with a domestic partner	5.3%
Weight status	Normal weight	17.9%
	Overweight	24.2%
	Obese	55.8%
	Underweight	2.1%
BMI		32.66 (9.11)
DEBQ		20.82 (15.59)
YFAS Severity (*n* = 68)		3.25 (2.05)
Food Addiction Status (*n* = 68)	Positive	22.1%
	Negative	77.9%
PCL		39.84 (20.78)
BDI		25.40 (14.08)

**Table 2:** Correlations between subjective social status in the society and the community with other variables of interest.

	Society	Community
Food Addiction Symptom Count (*n* = 68)	0.36 **	0.40 **
Dutch Eating Behaviors Questionnaire	0.21 *	0.30 **
Body Mass Index	0.15	0.29 **
PTSD Symptoms Severity	0.31 **	0.24 *
Depression Symptom Severity	0.45 **	0.29 **

Note: *n* = 89. Note: PTSD = posttraumatic stress disorder. * *p* < 0.05; ** *p* < 0.01.

**Table 3:** Correlations between objective social status indicators (i.e., highest level of education, current employment, and monthly household income) and the outcome variables.

	Education	Employment	Income
Food Addiction Symptom Count (*n* = 68)	−0.15 (*n* = 68)	0.09 (*n* = 67)	0.01 (*n* = 65)
Dutch Eating Behaviors Questionnaire	−0.05 (*n* = 90)	0.02 (*n* = 89)	−0.01 (*n* = 86)
Body Mass Index	−0.19 (*n* = 95)	−0.12 (*n* = 94)	0.01 (*n* = 91)

Note: *n* = 89.

**Table 4:** Hierarchical linear regression of perceived social status in the society and the community on emotional eating measured by the Dutch Eating Behavior Questionnaire, statistically adjusting for body mass index (BMI) and depression severity.

	*R* ^2^	*F*	Standardized Beta	*t*	*p*	Δ*R*^2^
Step 1	0.08	3.82			0.026	0.08
Constant				1.17	0.244	
BMI			0.12	1.17	0.246	
Depression severity			0.24	2.26	0.027	
Step 2	0.14	3.52			0.011	0.06
Constant				0.21	0.836	
BMI			0.07	0.69	0.494	
Depression severity			0.16	1.37	0.175	
Society social status			0.02	0.16	0.872	
Community social status			0.26	2.23	0.029	

**Table 5:** Hierarchical linear regression of perceived social status in the society and the community on food addiction severity, statistically adjusting for body mass index (BMI), posttraumatic stress disorder (PTSD) severity, and depression severity.

	*R* ^2^	*F*	Standardized Beta	*t*	*p*	Δ*R*^2^
Step 1	0.13	3.01			0.037	0.13
Constant				0.71	0.479	
BMI			0.15	1.25	0.216	
Depression severity			0.03	0.18	0.860	
PTSD severity			0.27	1.75	0.086	
Step 2	0.26	4.25			0.002	0.13
Constant				−0.31	0.755	
BMI			0.03	0.28	0.782	
Depression severity			−0.12	−0.72	0.476	
PTSD severity			0.25	1.68	0.099	
Society social status			0.21	1.51	0.137	
Community social status			0.30	2.24	0.029	

**Table 6:** Hierarchical linear regression of perceived social status in the society and the community on body mass index, statistically adjusting for sex and food addiction status.

	*R* ^2^	*F*	Standardized Beta	*t*	*p*	Δ*R*^2^
Step 1	0.09	2.97			0.059	0.09
Constant				8.91	<0.001	
Sex			0.16	1.24	0.218	
Food addiction			0.23	1.82	0.074	
Step 2	0.17	2.99			0.026	0.08
Constant				5.76	<0.001	
Sex			0.08	0.64	0.526	
Food addiction			0.14	1.11	0.273	
Society social status			0.05	0.37	0.715	
Community social status			0.28	2.04	0.046	

## Data Availability

The data are available upon request.
